# Supplementary material for: Molecular Characterisation and Phylogeny of Tula Virus in Kazakhstan
Source: Viruses. 2022 Jun 9;14(6):1258. doi: 10.3390/v14061258 (PMC9230364; doi:10.3390/v14061258)
Supplement: Supplementary file 1 [file viruses-14-01258-s001.zip › viruses-1704626-supplementary.pdf]

viruses

MDPI

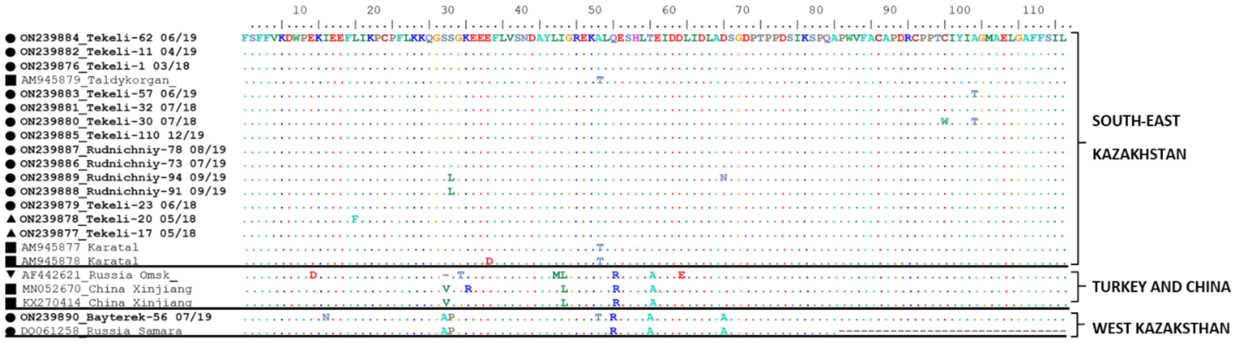

Figure S1. All available amino acid S segment sequences from Kazakhstan and close geographic regions in Russia (Omsk, Samara) and China.

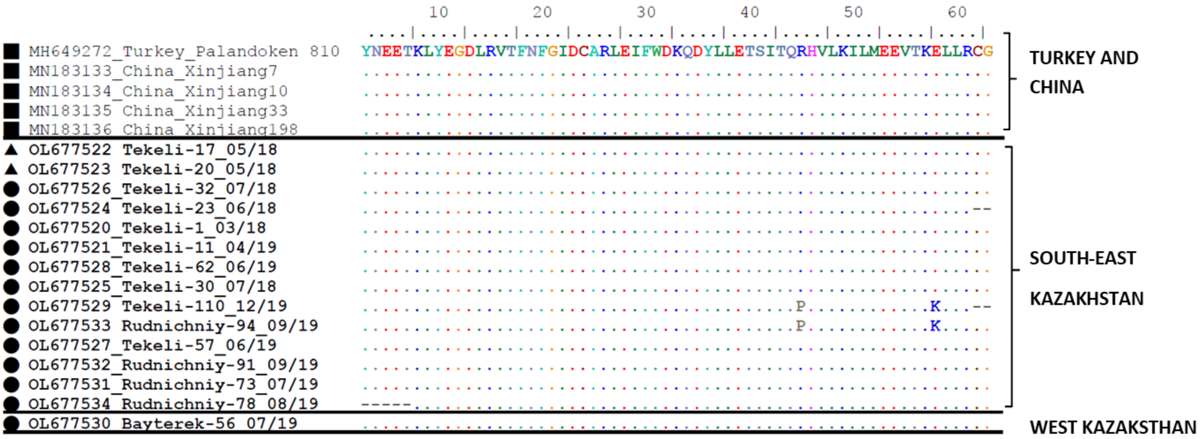

Figure S2. All available amino acid L segment sequences from Kazakhstan and close geographic regions in Russia (Omsk, Samara) and China.
